# 612. The Impact of Amputation on Psychiatric Outcomes After Burn Injury

**DOI:** 10.1093/jbcr/irag033.426

**Published:** 2026-04-06

**Authors:** Philong Nguyen, Aidan R Carroll, Joshua Wang, Yousef Tanas, Noelle Gonzalez, Kamryn K Baker, Luka Zrnic, Sudhanvan Iyer, Carolyn Henein, Jong Lee

**Affiliations:** University of Texas Medical Branch, Galveston, TX; University of Texas Medical Branch, Galveston, TX; University of Texas Medical Branch, Galveston, TX; Houston Methodist Hospital, Houston, TX; University of Texas Medical Branch, John Sealy School of Medicine, Houston, TX; McGovern Medical School at University of Texas Health Science Center at Houston, Houston, TX; University of Texas Medical Branch, Galveston, TX; University of Texas Medical Branch, Galveston, TX; University of Texas Medical Branch, John Sealy School of Medicine, Houston, TX; Division of Burn and Trauma Surgery, Department of Surgery, University of Texas Medical Branch, Galveston, TX

## Abstract

**Introduction:**

Amputation after burn injury is linked to significant psychological burden, including depression, anxiety, PTSD, and reduced quality of life. Prior studies are limited by small cohorts and short-term follow-up, leaving long-term psychiatric outcomes poorly defined. This retrospective cohort study uses a large multicenter database to quantify the psychiatric impact of burn-related amputation compared with matched individuals without limb loss.

**Methods:**

The TriNetX Research Network was queried for adult burn patients (≥18 years) hospitalized between 2003 and 2023. Two cohorts were identified using ICD-10 and CPT codes: patients who underwent amputation after burn injury and those without amputation. Propensity score matching (1:1) was performed based on demographics, body mass index, comorbidities, substance use history, preexisting psychiatric diagnoses, antidepressant use, and total surface burn area. Primary outcomes included new diagnoses of anxiety, depression, posttraumatic stress disorder (PTSD), adjustment disorder, self-harm, substance use disorders (SUD), and sleep disorders. Secondary outcomes included new antidepressant use. Patients with prior psychiatric diagnoses or antidepressant use before the study window were excluded. Outcomes were assessed 6 month, 1 year, and 2 year follow-up, with statistical significance set at *p*<.05.

**Results:**

After matching, 2361 patients were included in each cohort. At 6 months, burn survivors with amputation demonstrated significantly higher incidence of anxiety (11.03% vs 5.60%, HR 2.06, *p*<.001), depression (9.19% vs 5.36%, HR 1.75, *p*<.001), PTSD (1.72% vs 0.81%, HR 2.14, *p*=.012), substance use disorder (6.30% vs 4.06%, HR 1.57, *p*=.019), adjustment disorder (3.93% vs 1.65%, HR 2.59, *p*<.001), and antidepressant use (19.40% vs 11.23%, HR 1.73, *p*<.001). These trends persisted at 1 and 2 years for anxiety, depression, PTSD, adjustment disorder, and antidepressant use, though the association with substance use disorder was no longer significant beyond 6 months. Sleep disorders and self-harm showed no differences at any time point.

**Conclusions:**

Amputation after burn injury is associated with increased risk of psychiatric morbidity, underscoring the need for early screening, psychosocial support, and long-term mental health follow-up. Prospective studies are needed to clarify mechanisms and guide targeted interventions.

**Applicability of Research to Practice:**

Identifying the psychiatric vulnerability of burn survivors with amputation supports proactive screening, multidisciplinary care, and targeted mental health resources to improve recovery and quality of life.

**Funding for the study:**

This study was supported by foundation funding through the Institute for Translational Sciences (UL1 TR001439), funded by the National Center for Advancing Translational Sciences at the NIH.

